# The cross-sectional effects of ribbon arch wires on Class II malocclusion intermaxillary traction: a three-dimensional finite element analysis

**DOI:** 10.1186/s12903-021-01859-8

**Published:** 2021-10-06

**Authors:** Qin Xie, Duo Li

**Affiliations:** Department of Stomatology, Fujian Medical University Union Hospital, No. 29 of Xinquan Street, Gulou District, Fuzhou, 350001 China

**Keywords:** Finite element analysis, Orthodontic arch wire, Anchorage loss, Class II malocclusion, Intermaxillary traction, Ribbon wire

## Abstract

**Background:**

The application of intermaxillary traction is often accompanied by the unexpected movement of dentition, especially anchorage teeth. The aim of this study was to comprehensively compare the influence of cross-sectional shape of ribbon arch wires with edgewise and round wires on intermaxillary traction in Class II malocclusion treatment using FEA simulation.

**Methods:**

The dentofacial structure was simulated in finite element software. A retraction force of 1.5 N was applied to different cross-sectional orthodontic arch wires: a ribbon wire (0.025 × 0.017-in. and 0.025 × 0.019-in.), a rectangular wire (0.017 × 0.025-in. and 0.019 × 0.025-in.) and a round wire (Φ 0.018-in. and Φ 0.020-in.).

**Results:**

Among the three groups, ribbon wire (0.025 × 0.017-in. and 0.025 × 0.019-in.) exhibited the lowest displacement in the X-axis (12.61 μm and 12.77 μm, respectively) and Z-axis (8.99 μm and 9.06 μm, respectively). However, the 0.025 × 0.017-in. ribbon wire showed the highest Y-axis displacement. In the round wire group, Φ 0.020-in. wire displayed less rotation than Φ 0.018-in. wire, where the sagittal, frontal and occlusal rotation of Φ 0.020-in. wire was almost half of that of Φ 0.018-in. wire. The movement of the first molar region was intermediate between the ribbon arch group and the round wire group. Notably, the values of the 0.025 × 0.017-in. arch wire displacement, which were higher than those of any other group, peaked at 0.019 mm in the central incisor region with a spike-like shape. The deformation range of the Φ 0.018-in. wire group was the largest in this study.

**Conclusions:**

The cross-section of the arch wire influenced force delivery in Class II intermaxillary traction. With the same shape, a larger cross-sectional area led to less mandibular dentition movement. For the rectangular arch wire and ribbon arch wire groups, since the height and width were inverted, the vertical displacement of anchorage teeth in the ribbon wire group was reduced, but the possibility of buccal tipping in mandibular anterior teeth also increased.

## Backgrounds

Class II malocclusion, which is associated with a convex soft tissue profile caused by either mandibular retrognathism or excessive growth of maxilla, constitutes a significant diagnostic percentage of the patients seeking orthodontic treatment [1–4]. Among the various treatment modalities employed, intermaxillary traction generates a pulling force via ligatures and has been one of the most widely used techniques [5, 6]. Intermaxillary traction should be used with caution, especially for those patients with high-angle Class II malocclusion, since this approach is frequently accompanied by anchorage loss, namely, unexpected movement of the permanent mandibular molar [7].

Previous studies have characterized the geometry and size effects of orthodontic arch wires on the ultimate orthodontic treatment outcomes [8, 9]. Brackets and arch wires are the main components of fixed appliance systems, in which the arch wire serves as a means of originating and delivering force. Pandis et al. reported that the characteristics of wire were more pronounced than the ligating mechanism of brackets in force–deflection curves [10]. During orthodontic treatment, different cross-section arch wires can be chosen and sequenced based on the periodical goal [11]. The initial phase is often conducted with an undersized round cross-sectional arch wire to provide continuous lower forces and for fastening during the alignment process, whereas the larger edgewise arch wire is selected in the adjustment period to enhance torque effectiveness.

Compared with contemporary edgewise wires, the cross-section of ribbon arch wires represents an inversion of height and width. Historically, the ribbon arch, the first orthodontic appliance coupled with brackets, was considered the progenitor of edgewise appliances and earned popularity in the 1920s [12]. The utility of the ribbon arch has diminished since the introduction of edgewise wire. Although attempts have been made to increase ribbon arch usage, especially in lingual treatment, very few studies have involved ribbon wires in general orthodontic applications [13].

In addition, there is a paucity of data from investigations into the role that the cross-sectional shape plays in maxillomandibular traction. The reason for this lack of data could be the great challenge in identifying tooth movement and deformation under the coexistence of sophisticated anatomical structures and various orthodontic elements [14]. Finite element analysis (FEA), which is an engineering and mathematical method for reaction prediction, has been suggested to be a reliable method in orthodontic research through craniofacial complex remodelling [15, 16].

The aim of this study was to comprehensively compare the influence of the cross-sectional shape of ribbon arch wires with edgewise and round wires on intermaxillary traction in Class II malocclusion treatment using FEA simulation.

## Methods

The study protocol was designed in compliance with the Helsinki Declaration and approved by the ethical committee of the Fujian Medical University Union Hospital. Through medical history interviewing and oral examination, a 20-year-old healthy male volunteer with a healthy craniofacial structure and complete dentition with normal crown root ratio and a normal occlusion, was selected for this study. Before inclusion in this study, this volunteer signed an informed consent form.

The finite element (FE) model was based on the computed tomography (Discovery CT750HD, GE, Boston, USA) data, which referred to the sectional images of the volunteer from the upper rim of the condyle to the lower edge of the mandible in digital imaging and communications in medicine (DICOM) format. The CT data were collected and imported to the processing software (Mimics 17.0, Materialise, Leuven, Belgium) to further obtain a virtual model of the maxilla and mandible in Geomagic Studio (Raindrop Geomagic, Morrisville, USA). Along with the outer surface of the mandible, the periodontal ligament (PDL) was constructed at 0.25 mm thickness. The central area of alveolar bone was cancellous bone surrounded by 1.5 mm thick cortical bone.

**Fig. 1:**
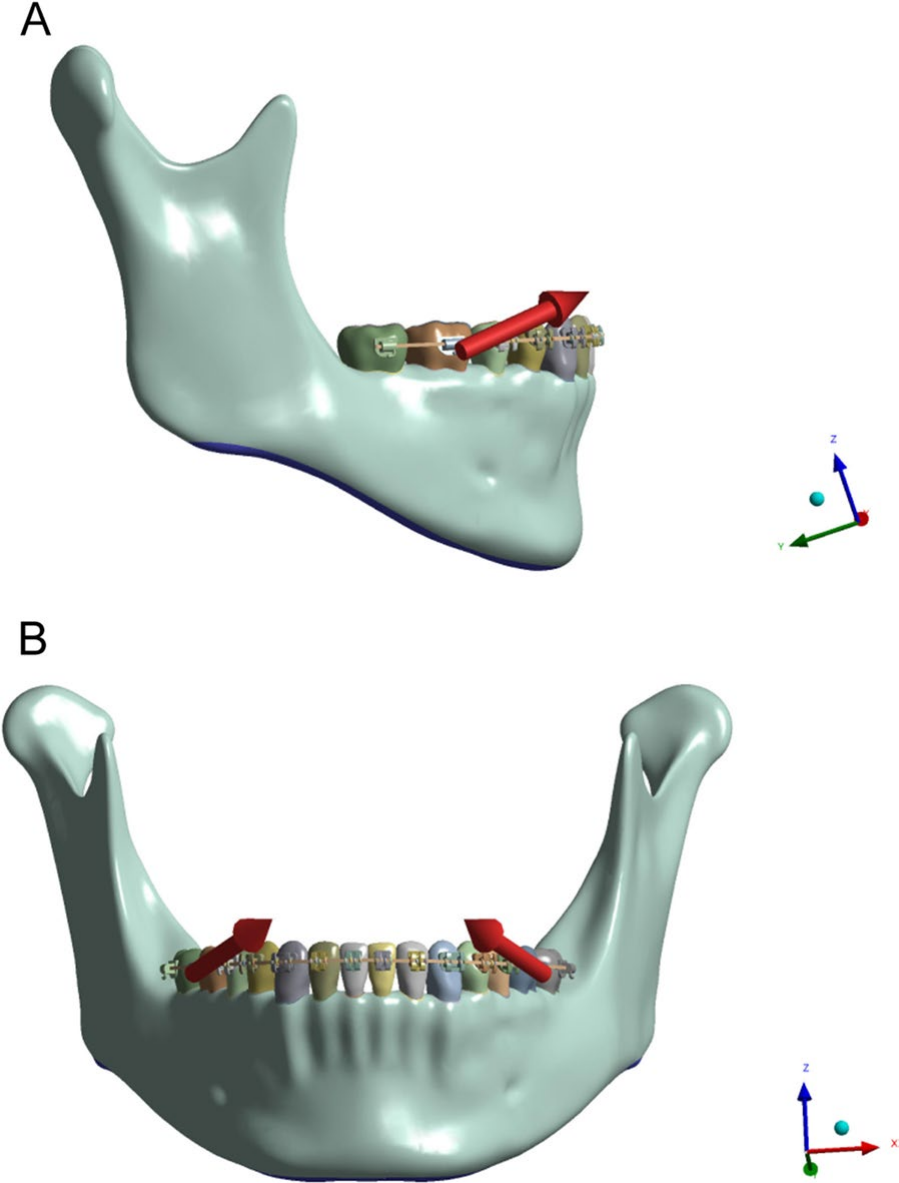
Side view and frontal view of dento-craniofacial simulation in the FEA with retraction force and the direction of intermaxillary traction force was indicated by red arrow

Rectangular wire (0.017 × 0.025-in. and 0.019 × 0.025-in., 3 M Unitek, Monrovia, USA) and round wire (Φ 0.018-in. and Φ 0.020-in., 3 M Unitek, Monrovia, USA) were scanned and these scans were entered into software (Unigraphics NX 8.5, Siemens PLM Software, Plano, USA). Using the same method, the matching brackets, buccal tubes and slots were modelled virtually. The ribbon wire (0.025 × 0.017-in. and 0.025 × 0.019-in.) was constructed based on the rectangular wire with height and width inverted. The rectangular wires and round wires were coupled with a 0.022 × 0.028-in. slot, whereas the ribbon wire was inserted into the 0.028 × 0.022-in. slot, which was designed for the purpose of fitting the upright arch wire. An anterior hook of 2.5 mm height was built and positioned between the maxillary lateral incisor and canine while the tubes were placed in the first mandible molar and second premolar. In addition, stainless steel ligature wires were tied to the brackets from the second premolar of the mandible right to the left second premolar. Those orthodontic appliances were assembled appropriately on the buccal side of dentition and are shown in Fig. 1.

A nonlinear elastic finite element analysis was performed in Ansys Workbench 15.0 (Ansys Inc., Canonsburg, USA). The Coulomb friction coefficient between the ligature and brackets was assumed to be 0.2 with reference to other experimental data. Each anatomical component and orthodontic appliance was simulated with ten-node tetrahedral elements and assumed to have isotropic homogenous linear elastic characteristics. The sizes of the elements in the PDL, tooth and bone were set as 0.5 mm, 1 mm and 2 mm, respectively, in order to simplify the finite element model, which included both small element size in the PDL and a large volume of the bone and the teeth. The FE model with different arch wires consisted of 616,006–650,197 nodes and 321,732–325,321 elements in total while the numbers of nodes/elements were 56,376/30,327 for teeth, 243,141/120,459 for the PDL, 199,834/111,827 for bone and 116,655–150,846/58,534–67,280 for the remaining components, including tubes, brackets and different arch wires. Young’s modulus and Poisson’s ratio of cortical bone, cancellous bone, teeth, PDL, arch wires, brackets and tubes were determined from previous studies [14, 17–23] and are shown in Table 1.

**Table 1:** Elasticity properties of materials in this study

	Poisson's ratio	Young's modulus (MPa)
Cortical bone [17, 18]	0.26	13,700
Cancellous bone [14, 17]	0.30	1370
Teeth [19]	0.30	19,600
PDL [19, 20, 23]	0.45	0.667
Tubes, brackets [21, 22]	0.30	206,000
Arch wire [21, 22]	0.30	176,000

A traction force of 1.5 N was applied from the maxilla anterior hook between the canine and lateral incisor to the hook of buccal tubes in the mandible first molar. The application of retraction force would initiate the movement of teeth, which could be defined by the displacement rotation of teeth, and the stress redistribution of the PDL. All the movement was suppressed for the nodes located in the bottom of the mandible segment.

There were two coordinate systems utilized in this study. For a single tooth, the x-axis, was referred to the mesio-distal direction (+ mesial, − distal), the y-axis referred to the buccal–lingual direction (+ lingual, − buccal) and the z-axis to the vertical direction (+ apical, − occlusal). For other large volume components (mandible, dentition, PDL and buccal/lingual line), the displacement of teeth was recorded on the basis of a standard coordinate system with the x-axis as the anterior–posterior direction, the y-axis as the medial–lateral direction, and the z-axis as the superior–inferior direction, whereas the anterior, medial, and superior directions were defined as the + x, + y, and + z directions, respectively. Rotation movement was decomposed into three planes of motion (sagittal, frontal and occlusal), and the corresponding directions (posterior, medial and mesial) were predetermined as positive.

The buccal cusps of the mandibular posterior teeth and the edge of anterior teeth were lined virtually as buccal lines, while the lingual cusps of mandibular posterior teeth and the cingulum of anterior teeth were connected as lingual lines. The movement of the lingual line and buccal line was recorded.

## Results

### Displacements of anchorage teeth

The results of 46 teeth in terms of force component distribution, displacement and rotation movements in three directions are shown in Table 2. Regarding the force distribution of 46 teeth, very similar results were found for the 0.017 × 0.025-in. and 0.019 × 0.025-in. wires. The force components of the X-axis, Y-axis and Z-axis were 1.36 N, 0.31 N and 0.45 N, respectively. Compared with the rectangular wire and round wire groups, the ribbon wire group had the lowest force component in three directions, and the force of the 0.025 × 0.017-in. group in the X-axis, Y-axis and Z-axis was 1.31 N, 0.29 N, 0.43 N, respectively. The round wire group displayed the highest X-axis force and Z-axis force (1.39 N and 0.48 N, respectively).

**Table 2:** The results of 46 teeth in terms of force component distribution, displacement and rotation movements in three directions

Group	Size	Force(N)			Displacement (μm)			Rotation(°)		
		X-axis	Y-axis	Z-axis	ΔX	ΔY	ΔZ	Sagittal	Frontal	Occlusal
Rectangular wire	0.017 × 0.025-in.	1.36	0.31	0.46	13.44	4.63	9.17	− 0.002	0.020	0.021
	0.019 × 0.025-in.	1.36	0.31	0.45	13.45	4.58	9.13	− 0.002	0.020	0.020
Ribbon wire	0.025 × 0.017-in.	1.31	0.29	0.43	12.61	6.50	8.99	− 0.003	0.042	0.045
	0.025 × 0.019-in.	1.31	0.30	0.42	12.77	4.95	9.06	− 0.003	0.025	0.026
Round wire	Φ 0.018-in.	1.39	0.29	0.48	13.76	5.96	9.44	− 0.002	0.034	0.036
	Φ 0.020-in.	1.39	0.29	0.48	13.81	4.40	9.20	− 0.001	0.016	0.017

For the rectangular wire group, with increasing wire size, there was a descending trend in the displacement in the Y-axis and Z-axis. The displacement of the 0.017 × 0.025-in. wire in the Z-axis direction was 9.17 μm and that of the 0.019 × 0.025-in. wire was 9.13 μm. Among the three groups, ribbon wire (0.025 × 0.017-in. and 0.025 × 0.019-in.) exhibited the lowest displacement in the X-axis (12.61 μm and 12.77 μm, respectively) and Z-axis (8.99 μm and 9.06 μm, respectively). However, the 0.025 × 0.017-in. ribbon wire showed the highest Y-axis displacement. The Φ 0.018-in. round wire also showed higher Z-axis displacement (9.44 μm) and Y-axis displacement (5.96 μm) than Φ 0.020-in. round wire (9.20 μm and 4.40 μm, respectively).

In the rectangular wire group and ribbon wire group, the rotation motion for 46 teeth in the sagittal, frontal and occlusal planes was − 0.002°, 0.020°, and 0.021°, respectively. In the ribbon wire group, the frontal rotation was higher than that in the rectangular and round wire groups, especially the 0.025 × 0.017-in. group, which exhibited 0.042° of rotation. In the round wire group, Φ 0.020-in. wire displayed less rotation than Φ 0.018-in. wire, where the sagittal, frontal and occlusal rotation of Φ 0.020-in. wire was almost half that of Φ 0.018-in. wire.

### von Mises stress distribution of the mandibular PDL

**Fig. 2:**
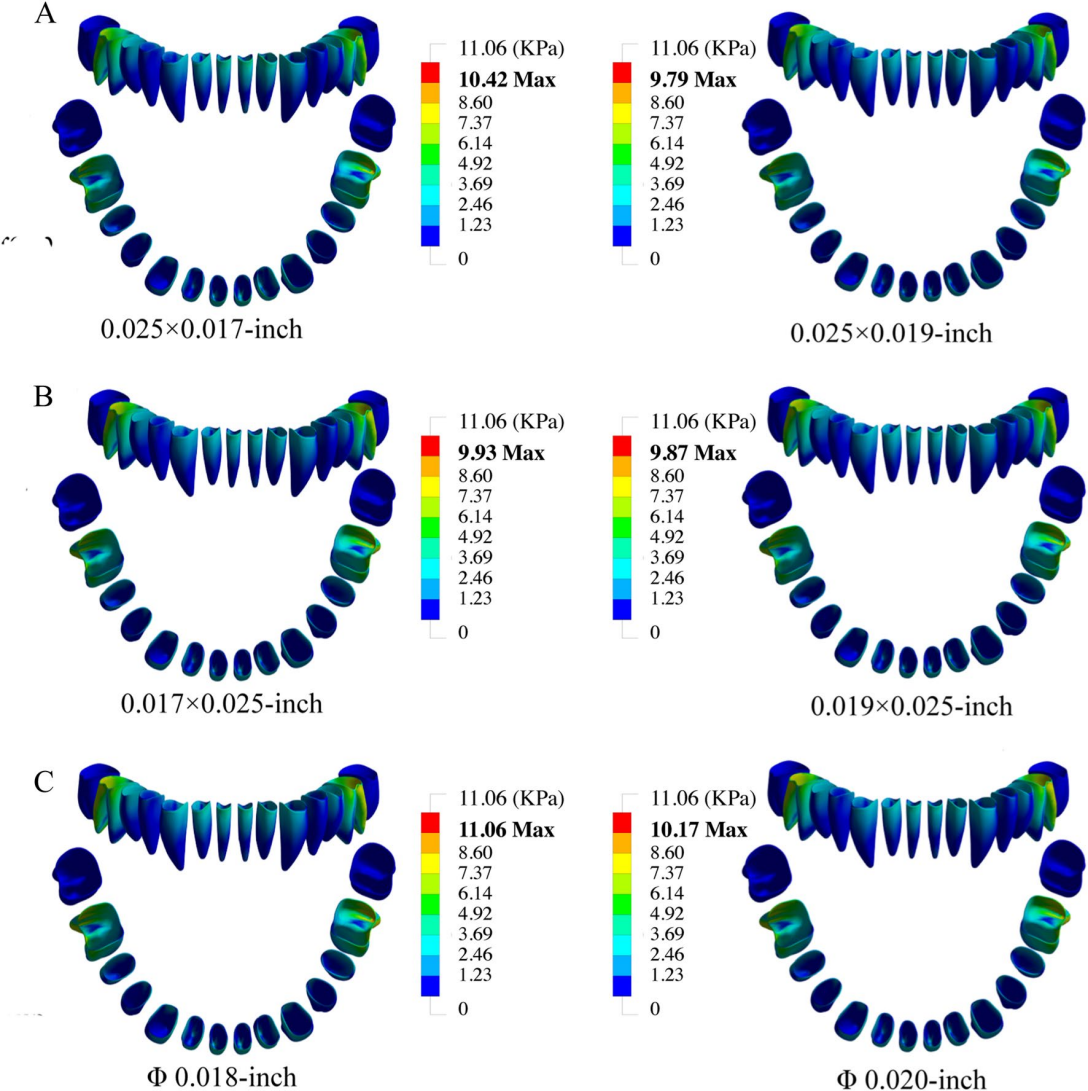
The frontal view and occlusal view of the PDL von Mises stress distribution of different groups. (**A** ribbon arch wires; **B** rectangle arch wires; **C** round arch wires) while the values of maximum stress in each group were indicated in bold

Figure 2 illustrates the buccal view and occlusal view of the PDL von Mises stress distribution. In the posterior zone, the PDL of the first molar endured the most stress, especially in the distal root, and stress decreased gradually in the mesial direction. In the anterior zone, there was an increasing trend in stress from the canine tooth towards the central incisor. As shown in Fig. 2, there was a very slight difference caused by the cross-sectional area in the PDL stress distribution among the study groups, and the maximum stress was was in the range of 9.87–11.06 kPa.

### Displacements of mandible dentition

**Fig. 3:**
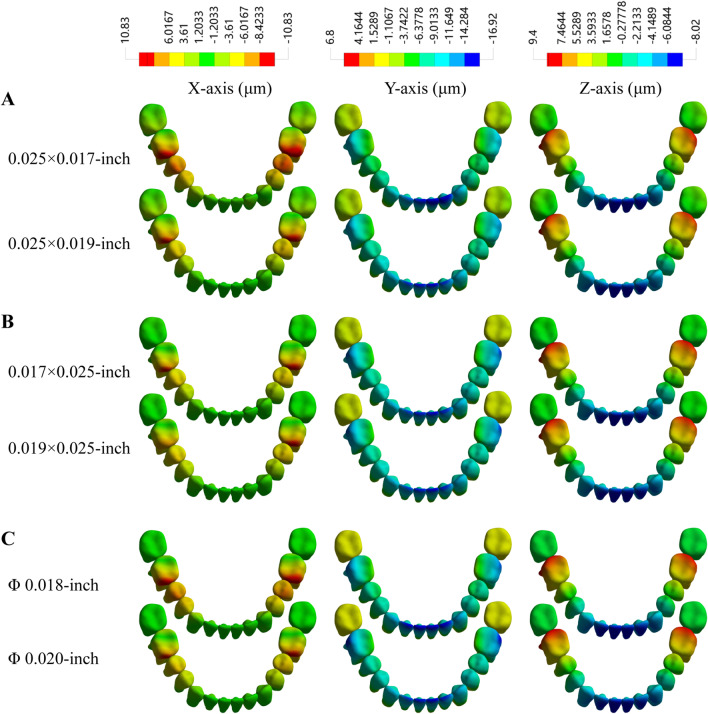
Displacements of the mandible dentition in X-axis, Y-axis and Z-axis (**A** ribbon arch wires; **B** rectangle arch wires; **C** round arch wires)

The detailed displacements (X-axis, Y-axis and Z-axis) of mandible teeth after the application of 1.5 N retraction force is shown in Fig. 3. In the X-axis direction, all the teeth were inclined mesially due to the retracting force. As the cross-sectional area enlarged, the displacement of mandibular dentition in the X-axis was reduced in ribbon wire group and rectangular wire group and round wire group. In the Y-axis direction, the posterior teeth were tipped to the lingual side, whereas the anterior teeth exhibited a labial-oriented motion. In the Z-axis direction, the first molar moved in an upward direction, but the central incisor had an opposite trend, indicating that the anterior teeth might intrude vertically since the posterior teeth tilted inward. The largest vertical displacement among mandible dentition was exhibited by the mandibular first molar, where the ribbon wire group exhibited the least vertical movement and the round wire group showed the highest displacement.

### Displacement of the buccal line and lingual line

**Fig. 4:**
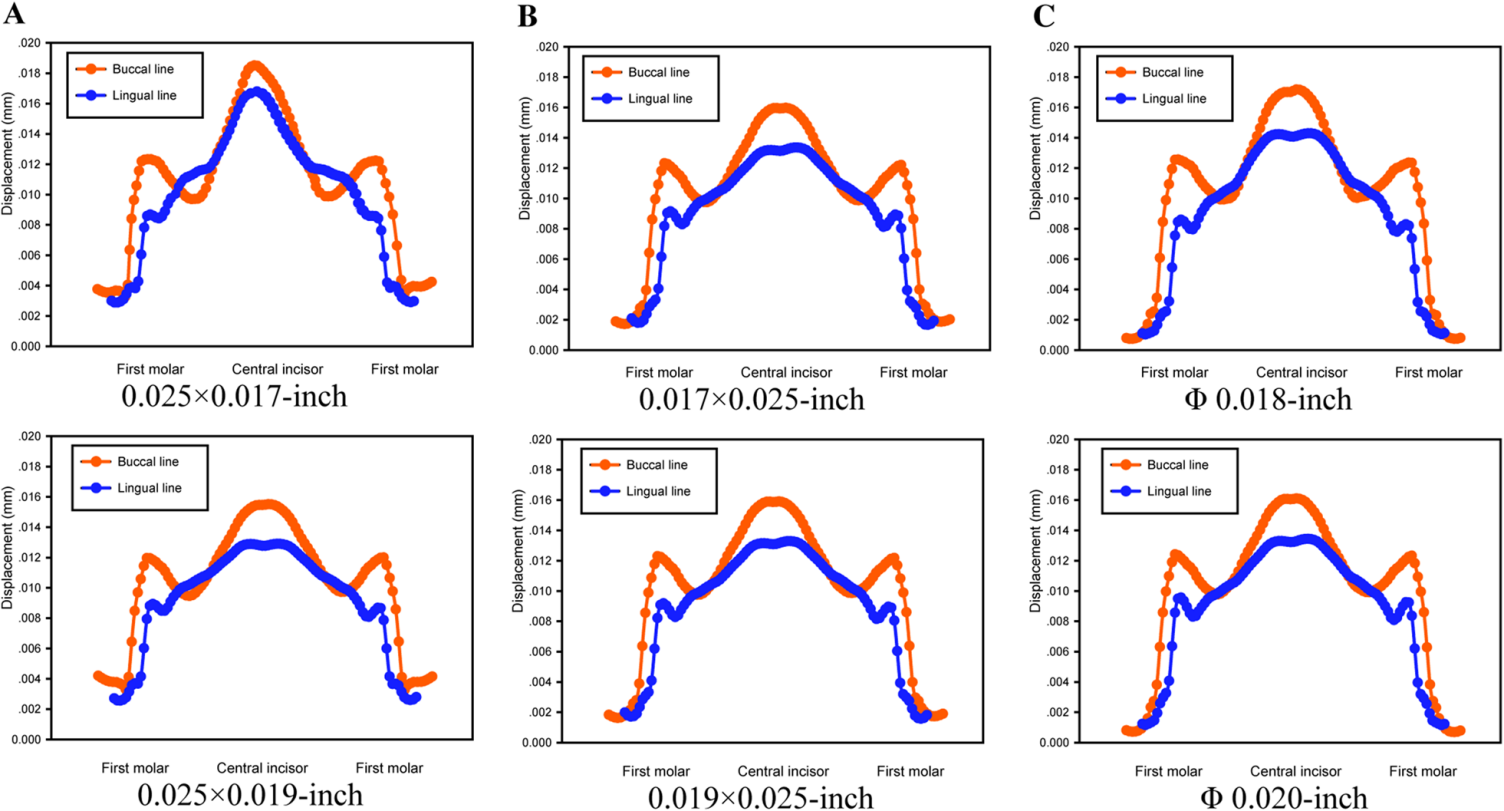
The buccal line (marked in red) and lingual line (marked in blue) displacements in different groups (**A** ribbon arch wires; **B** rectangle arch wires; **C** round arch wires)

The displacements of the buccal line and lingual line in different groups are shown in Fig. 4 (red refers to the buccal line, and blue refers to the lingual line). From the observation of the curve contour, the tendencies of the buccal line and lingual line in different arch wire groups showed some similarity. The displacement was dramatically increased in the first molar region, followed by a slight decrease in the premolar zone before subsequently rising in the anterior teeth region.

In the rectangular wire group (0.017 × 0.025-in. and 0.019 × 0.025-in.), the maximum displacements of the buccal line and lingual line were 15.98 μm and 15.91 μm, respectively. The movement of the first molar region was intermediate between the ribbon arch group and the round wire group. In the ribbon arch group, the lingual line displacement in the first molar region was 0.008 mm, which was also the lowest among the corresponding results of all the studied groups. Notably, the values of the 0.025 × 0.017-in. arch wire displacement, which were higher than those of any other group, peaked at 18.55 μm in the central incisor region with a spike-like shape. In the 0.025 × 0.019-in. group, the displacements ranged from the first molar to the central incisor of the buccal line, and the lingual line was lowest among the studied groups. In the round wire group, the displacement ranging from the first molar to the central incisor of the buccal line and lingual line was higher than those of the rectangular and ribbon arch groups. The movement induced by the Φ 0.020-in. wire was 16.11 μm, which was lower than that induced by the Φ 0.018-in. wire (17.18 μm).

### Deformation of the arch wire

**Fig. 5:**
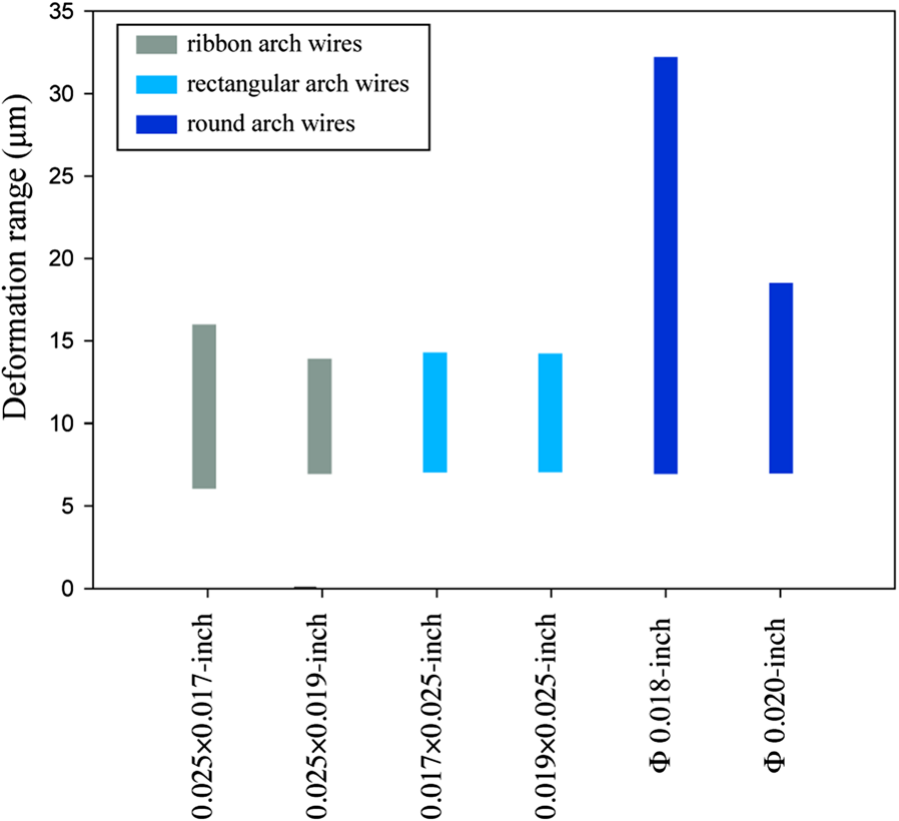
The deformation range between maximum deformation and minimum deformation in different groups after the application of 1.5 N retraction force

The deformation range between maximum deformation and minimum deformation in different groups after the application of 1.5 N retraction force is summarized in Fig. 5. The rectangular wire group and ribbon wire group displayed a relative stress concentration in the anterior zone. The deformation range of the Φ 0.018-in. wire group was the largest (25.27 μm) in this study while the deformation range in 0.025 × 0.019-in. group was the lowest (6.94 μm).

## Discussion

The high prevalence of Class II malocclusion in patients prompted the wide usage of intermaxillary elastic traction. However, scarce data and few documented details are available regarding the influence of arch wire geometry during the maxillomandibular traction procedure. In the present study, we principally aimed to simulate the dentofacial structure via FEA to explore the effect of arch wire cross-sectional shape on Class II intermaxillary traction.

Often, the changes in vertical and horizontal vectors caused by Class II intermaxillary traction involve anchorage loss [7, 24, 25]. There was an average growth of 5.0 mm of the lower face height after an average duration of 1.3 years for the fixed appliances and Class II elastics, with which mandibular growth could surpass maxilla growth by 1.1 mm [4]. Since maxillary growth was suppressed by intermaxillary traction, Jason et al. observed a 1.2 mm mesial movement of the mandibular first molar [5]. Nina el al. found that there was an open bite in half of the high-angle Class II patients who received bimaxillary surgery and mandibular skeletal relapse in 40% of the patients [26]. The adverse effect of anchorage loss tended to clockwise rotation of the occlusal plane and aggravate the treatment difficulty of high-angle Class II malocclusion.

Based on the FEA model in this study, differential results were observed in the arch wire group with different cross-sections in terms of mandibular first molar movement. The mechanics of arch wires have been recognized as one of the contributing factors of anchorage loss but have not been fully investigated [5]. Theoretically, the moment of inertia (*I*) of the rectangular wire is equal to “h^3^w/12” (where “h” refers to the height of the cross-section and “w” refers to the width); *I* of the round wire is equal to “πr^4^/64” (where “r” refers to the radius of the cross-section) [11]. When the diameter of the circular wire is enlarged by 20%, the bending stiffness is slightly more than doubled [27]. In addition, the magnitude of the height in the cross-section of the rectangular wire was far greater than the width. The formula indicated the possibility of creating a more rigid rectangular wire by only changing only height and without changing the total area. In this study, the ribbon wire group exhibited the lowest vertical and horizontal displacement, followed by the rectangular wire group and round wire group.

In this FE study, the remarkable execution of the ribbon wire in minimizing the vertical and horizontal displacements provided the basis for reviving the ribbon wire. The good performance of the ribbon wire in anchorage control could be attributed to the relatively greater height of the arch wire cross-section. A greater height tended to enlarge the moment of inertia and provide more rigid support to the occlusal plane. To our knowledge, only Inami et al. reported a minimum vertical bowing effect with a ribbonwise lingual appliance [28]. However, they also remarked on insufficient control of the ribbon wire in lingual tipping due to the thinner geometry, and a palatal bar was needed to keep the tooth upright. The drawback was consistent with the FEA results in this study since the ribbon wire group displayed greater Y-axis displacement and frontal rotation than other groups.

There were very similar results displayed by different arch wire groups in which the PDL around the distal root endured the most stress. Previous studies also revealed the relatively high occurrence of root resorption in the distal root of the mandibular first molar in orthodontic treatment [29]. In the present FE study, the elastic characteristics of the PDL were assumed to be isotropic homogenous linear elastic, rather than the nonlinear viscoelastic, to facilitate the FE reconstruction process. After testing 4 different FE modelling strategies, Hohmann et al. has pointed out that the PDL is insensitive to the modelling and reconstruction techniques for low orthodontic forces [30]. Xu et al. combined the optical measurements and numerical simulations to determine the elastic modulus of the PDL and they reported a range from 0.01 to 0.08 MPa (0.04 ± 0.02 MPa) in the initial elastic phase [31]. Therefore, the elastic modulus (E = 0.667) in this study could be interpreted as acceptable.

The displacement results of the buccal line and lingual line as well as the displacements of mandibular dentition also demonstrated that there was an association between cross-sectional geometry and mandibular tooth movement in Class II intermaxillary traction. Monlasser et al. stated that the importance of the arch wire cross-section in mechano-therapy should be appreciated since the force level in the vertical direction was amplified from 16 to 120% when the 0.014-in. arch was replaced by the 0.016-in. [32]. In the present study, with the same shape, a larger cross-sectional size would resulted in mandibular dentition movement. The increasing interspace between the bracket and arch wire induced less friction and less slack in the root controls, which might be detrimental to the en masse retraction of maxillary anterior teeth [33]. Similarly, the displacement ranges from the first molar to the central incisor of the buccal line and lingual line were higher than those of the rectangular and ribbon arch groups.

Arch deformation is directly correlated with the resistance to sliding, and permanent wire deformation heavily encumbers tooth movement efficiency [34]. From the perspective of the second moment of inertia, the ribbon arch wire group (0.025 × 0.017-in. and 0.025 × 0.019-in.) had greater bending resistance than the rectangular wire group (0.017 × 0.025-in. and 0.019 × 0.025-in.) since the width and height were inverted. Compared with that of the rectangular wire group, the vertical displacement of anchorage teeth in the ribbon wire group was reduced, but the possibility of buccal tipping of mandibular anterior teeth was also increased. The 0.025 × 0.017-in. ribbon arch wire group showed a spike-like shape peak in the central incisor area, indicating inferior control of the mandibular anterior region, as mentioned previously. Studies have concluded that round arch wires exhibit lower friction than rectangular wires [35, 36]. However, in this study, the round arch wire, especially the Φ 0.018-in. wire, displayed greater arch deformation than the edgewise groups. The reason might be ascribed to the relatively lower bending stiffness of the round arch wires. The wire deformation of the rectangular arch was close to that of the ribbon arch group.

There were some limitations in this study because this experiment was conducted entirely using computer software. For instance, the setting of biological characteristics may differ from the real properties in vivo. Future studies, especially clinical trials, should be conducted to investigate the effect of arch wire geometry in vivo.

## Conclusions

Within the limitations of this FEA, the cross-section of the arch wire exerted an influence on force delivery in Class II intermaxillary traction. With the same shape, a larger cross-sectional size led to smaller mandibular dentition movement. For the rectangular arch wire and ribbon arch wire groups, since the height and width were inverted, the vertical displacement of anchorage teeth in the ribbon wire group was reduced, but the possibility of buccal tipping of mandibular anterior teeth also increased.

## Data Availability

The datasets used and/or analysed during the current study are available from the corresponding author on reasonable request.

## Abbreviations

FE: Finite element
CT: Computed tomography
PDL: Periodontal ligament
